# Effects of 16-Day Fluoxetine and pCPA Exposure on the Velocity of the Invasive Crayfish *Procambarus clarkii*

**DOI:** 10.3390/biology15181670

**Published:** 2026-09-21

**Authors:** Daniel Oliveira, Filipe Banha, Pedro Anastácio, Marta C. Soares

**Affiliations:** 1 MARE—Marine and Environmental Sciences Centre/ARNET—Aquatic Research Network, Universidade de Évora, 7004-516 Évora, Portugal; daniel.f.a.oliveira4694@gmail.com (D.O.); filipebanha@uevora.pt (F.B.); 2 Institute for Research and Advanced Training (IIFA), Universidade de Évora, 7004-516 Évora, Portugal; 3 MARE—Marine and Environmental Sciences Centre/ARNET—Aquatic Research Network, Departamento de Paisagem, Ambiente e Ordenamento, Universidade de Évora, 7004-516 Évora, Portugal; anast@uevora.pt; 4 CIBIO, Research Centre in Biodiversity and Genetic Resources, InBIO Associate Laboratory, Vairão Campus, University of Porto, 4485-661 Vairão, Portugal; 5 BIOPOLIS Program in Genomics, Biodiversity and Land Planning, CIBIO, 4485-681 Vairão, Portugal; 6 CIBIO, Research Centre in Biodiversity and Genetic Resources, InBIO Associate Laboratory, School of Agriculture, University of Lisbon (ISA), 1349-017 Lisboa, Portugal

**Keywords:** selective serotonin reuptake inhibitor (SSRI) fluoxetine, locomotion, *Procambarus clarkii*, invasive species

## Abstract

Fluoxetine, a selective serotonin reuptake inhibitor (SSRI), is one of the most detected antidepressants in wastewater, managing to enter watersheds where it is taken up by freshwater fauna. Here we asked if the serotonergic system affects the locomotive capabilities of *Procambarus clarkii*, a prolific worldwide invasive crayfish species, thanks in part to its dispersal rates. For this, we exposed adult crayfish to 1 µg/L and 100 µg/L solutions of fluoxetine and para-chlorophenylalanine (pCPA, a tryptophan hydroxylase inhibitor) and measured their velocity after 8 and 16 days on a two-metre-long metal gutter. Overall, our treatment effects were revealed to be non-significant, suggesting *P. clarkii* has high tolerance to fluoxetine and pCPA and that at environmentally significant concentrations fluoxetine may not affect *P. clarkii* locomotion. Though our study was of limited scope and further research is needed with revised set-ups, these results can help us better understand the impact of ambient fluoxetine on this invasive species.

## Simple Summary

Fluoxetine is one of the most detected antidepressants in freshwater environments where it is taken up by fauna. Here we tested if serotonin affects the dispersal capabilities of the invasive crayfish *Procambarus clarkii* by exposing adults to two concentrations of fluoxetine and a pCPA for 8 and 16 days. Results have not shown any significant changes in locomotion between treatments except. Follow-up studies with revised designs are suggested.

## 1. Introduction

Fluoxetine is one of the most prescribed selective serotonin reuptake inhibitors (SSRIs) in western countries. Similarly to other drugs that are excreted or discarded by humans, fluoxetine passes unchanged through wastewater treatments and enters natural waterways [1], making it historically the most detected antidepressant in wastewaters [2]; its concentrations in freshwater even exceed environmentally relevant levels (<100 ng/L), going as high as 330 ng/L (North Carolina, USA) [3]. Fluoxetine acts acutely by elevating the levels of the neuromodulator serotonin (5-HT) in synapses. This neuro-endocrine system is evolutionarily conserved both in vertebrates and invertebrates [1] and modulates several functions [4]. In crustaceans, serotonin modulates tonic discharge of motoneurons and enhances synaptic transmission in neuromuscular junctions [5,6] and has anxiogenic properties [7,8] reducing exploration. This also affects risk assessment, increasing or decreasing aggressive behaviours [9].

Though acute effects are uncommon, ambient fluoxetine can be taken up and stored in animal tissues [10,11]. When chronically exposed to fluoxetine, it can elicit changes in the release of serotonin and the expression of serotonin receptor genes and alter the activity of other transmitter systems [12,13]. The metabolism of circulating biogenic amines, namely serotonin, may change from species to species; for instance, in the crayfish *Orconectes limosus*, the half-life of serotonin in the haemolymph is relatively short (which averages 12.5 min and 13.5 min, respectively, at 15 °C); however, 20–25% ends up in important tissues: green gland, digestive gland and gills [14]. However, compared to the immediate effects of serotonin, the absorption, tissue accumulation and half-life of fluoxetine may take up to 4 to 15 days [15]. In the shore crab *Carcinus maenas* high concentrations of fluoxetine were observed in the digestive gland, an organ primarily responsible for xenobiotic detoxification [16] and in brain/nervous tissues [17]. These authors observed that fluoxetine can remain in crabs’ tissues for more than 10 days, confirming the compound’s high bioaccumulation potential and efficient biotransformation [17]. These findings may also be relevant to other invertebrates’ species and suggest that even low-level environmental exposure could result in measurable concentrations within the organism, especially in brain tissue, and potentially affect behaviour. Indeed, behavioural changes following exposure to fluoxetine have been reported in many aquatic species, such as fish [18,19,20] and crustaceans [21,22,23].

One important variable that fluoxetine may affect is an organism’s mobility [21,22,23]. In fish species, exposure to fluoxetine can decrease swimming activity. For example, Duarte et al. [19] observed a decrease in *Pomatoschistus microps* active time and an increase in movement delay at concentrations of 1 and 5 mg/L, and Meijide et al. [20] observed that *Gambusia holbrooki* had longer motionless periods at concentrations of 25 μg/L and 50 μg/L. Similar observations were made in crustaceans, with Tierney et al. [1] observing that juvenile *Orconectes rusticus* exposed for 7 days to concentrations of 2 and 500 µg/L of fluoxetine dissolved in water reduced their locomotion compared to controls. However, fluoxetine can also have the opposite effect, increasing swimming activity, as observed with *Aphanius dispar* juveniles when exposed to 0.03 and 0.3 μg/L fluoxetine for 7 days [24].

Also, Pan et al. [25] observed an increase in swimming distance and velocity in *Danio rerio* larvae exposed to 100 μg/L of fluoxetine for 5 days. Additionally, Vera-Chang et al. [26] reported an increase in the locomotor activity of adult *Danio rerio* exposed to 0.54 μg/L for 6 days. Similarly in crustaceans, Mesquita et al. [23] exposed *Carcinus maenas* to concentrations of fluoxetine greater than 120 µg/L for 10 days and showed that locomotion was significantly increased with animals moving for longer periods of time and walking further.

Similarly to other aquatic species, invasive crayfish species locomotion might also be affected by fluoxetine, but no studies currently address this critical issue. This gap is significant since the impact and success of invasive species often depend on their dispersal ability and subsequent ecological effects [27]. One of these species is the red swamp crayfish, *Procambarus clarkii*, an omnivorous freshwater crayfish of the Cambaridae family, native to northeastern Mexico and the southern USA. This polytrophic keystone species can exert multiple pressures on ecosystems and is a prominent example of a successful invasive species with strong individual variations in dispersal distances [28,29] having been introduced on all continents apart from Antarctica and Oceania. It is currently recorded in 16 European countries [29], and it is considered the most ubiquitous freshwater crayfish species in the world [30,31,32,33]. Its success as an invasive species stems from characteristics such as early maturity, a fast growth rate, a high offspring number, a rapid and adaptable life cycle, a flexible feeding strategy, and a wide ability to disperse and to tolerate extreme environmental changes [29,34,35] such as in temporary streams (i.e., in southern Portugal [36]) and polluted habitats [37].

Due to its invasive nature, understanding of how *P. clarkii* motor activity may be modulated by the serotonergic system is key to predicting its potential spread and may help formulate new plans and strategies to mitigate its spread. As such, the aim of this study is to test the effects of changing exogeneous fluoxetine exposure on crayfish motor activity. To achieve this, we measured the velocities of *P. clarkii* individuals as a proxy for locomotive activity after these have been exposed for 16 days to two concentrations of fluoxetine as well as para-chlorophenylalanine (pCPA), a tryptophan hydroxylase inhibitor that acts as a potent depletor of tissue serotonin in animals [38] to also test the effects of lowering serotonin levels [39]. Considering previous studies on crayfish as well as other teleost fish species, we expected that exposure to fluoxetine would lead to crayfish velocity decrease, whereas we expected it to increase with pCPA exposure due to its antagonistic nature to fluoxetine.

## 2. Materials and Methods

### 2.1. Crayfish Capture and Housing

*Procambarus clarkii* individuals were captured in the Marateca stream next to rice fields near the town of Cabrela, Portugal (38.58561 N, 8.528580 W). These were captured on two separate occasions with spiral nylon net traps (60 cm × 32 cm, 10 mm mesh) baited with sardines and left for around 20 to 24 h overnight. One catch was between 11 and 12 May 2023 and a second between 13 and 14 July 2023. These catches yielded only adults, with no individual below 25 mm of cephalothorax length. This was most likely due to trap catches being biased towards capturing larger individuals [40].

Captured crayfish were maintained collectively inside four rectangular holding tanks (50 × 100 × 45 cm) in an acclimatised room at 20 °C until they were singled out and identified. The tanks were filled with a depth of 7 cm of regular tap water, enough to cover the crayfish completely, and aerated using an aquarium pump under a 12 h light/12 h dark cycle. 6 to 8 PVC tube sections were scattered on the bottom of the tanks to serve as shelter. Crayfish were fed carrots while in the tanks, both as a lower-cost but still nutritious alternative to the sardines used during capture. It also prevented fighting amongst crayfish that often clash to access the carcass. Every four to seven days crayfish were removed temporarily to clean the containers, replace the water and replenish food items. While crayfish from the first catch were housed in these tanks for close to 4 months, those from the second catch were only housed for 2 weeks. We accounted for this when distributing crayfish through the 5 different treatments.

Crayfish wet weight and cephalothorax length were measured and used to calculate their physical condition using Fulton’s condition index with the formula K = W/L^3^, where W is wet weight (g) and L is Cephalothorax length (mm) [41]. These values were then used to distribute the individuals into groups with similar size, weight and physical condition. The selected crayfish were then kept individually inside small containers with 300 mL of internal volume and filled with 200 mL of water for a minimum of 5 days before being exposed to the designated solutions. This 5-day interval was to account for the two different housing periods that the crayfish from the two catches underwent. The top covers of the containers were perforated, allowing an appropriate volume of open-air space to properly oxygenate the water. The containers were cleaned and the water and uneaten food replaced with fresh water and food every two days. During the entire housing period, crayfish were fed carrots as before.

### 2.2. Experimental Design and Physiological Treatments

**Figure 1:**
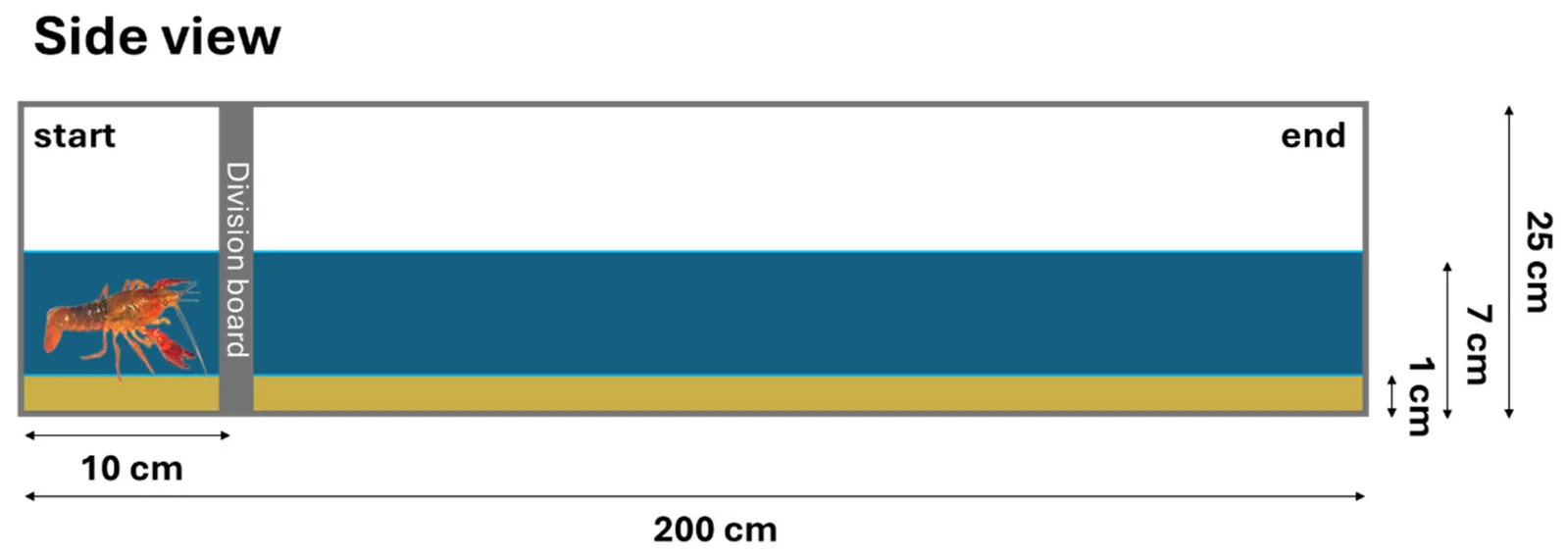
Schematic side view of the general layout used for the experiments.

The setup consisted of a two-metre-long metal gutter, the bottom of which was covered with 1 cm of medium coarse sand (0.5 to 1 mm grain) and filled with a depth of 7 cm of tap water. A removable translucent plastic plate separated the initial 10 cm of the track, where crayfish are initially placed, from the rest of it (Figure 1).

We used 144 adult crayfish in this experiment, with a mean cephalothorax length of 45.01 mm (sd = 3.67 mm). There were 85 males and 59 females, seven of which were carrying eggs at the time of the experiments. The berried females were distributed as evenly as possible through the five treatments; however, two of the females laid eggs during the first exposure period, resulting in 3 berried females in the fluoxetine 1 µg treatment and 1 per every other treatment.

The crayfish were assigned into 5 treatments balanced for size and physical condition through stratified randomisation, with 29 individuals each, except the 1 µg/L fluoxetine treatment, which had 28 crayfish due to the removal of one individual that had difficulties in submerging during the experiment.

Each crayfish was immersed in 200 mL of solutions of either 1 µg/L of fluoxetine, 100 µg/L of fluoxetine, 1 µg/L of pCPA or 100 µg/L of pCPA. The fifth group acted as a control and was immersed in 200 mL of regular tap water. The mean cephalothorax length for the fluoxetine 1 µg/L was 45.04 mm (sd = 3.758); for the fluoxetine 100 µg/L it was 45.00 mm (sd = 3.332); for the pCPA 1 µg/L it was 44.98 mm (sd = 4.15); for the pCPA 100 µg/L it was 44.97 (sd = 3.766); and for the control group it was 45.04 mm (sd = 3.398).

The lowest concentration used, which was higher than the maximum freshwater levels of fluoxetine detected [3], was chosen in the expectation that an effect might be observed. The 100 µg/L was chosen to give a noticeably distinct order of magnitude. pCPA at the same concentrations was used to test the effects of serotonin disruption, the opposite effect of fluoxetine. Though there is no evidence that fluoxetine and pCPA elicit similarly strong responses, pCPA has been shown to reduce serotonin levels in crayfish [39]. Due to this, we hoped to observe an opposite effect to that of fluoxetine to help us better understand how the serotonergic modulates locomotion in crayfish.

Crayfish were exposed to the solutions for 16 days, with experiments conducted on the 8th and 16th days, giving a total of two days of experiments. The solutions and food items were replaced with new solutions and food every three days. The metal track was emptied and cleaned along with the sand substrate between the two experiment days.

### 2.3. Behavioural Analysis

Crayfish went through 3 days of fasting and were acclimatised to a temperature of 22 °C for 24 h before the day of the experiment. The temperature used in this experiment corresponds to water temperatures registered in spring and summer in rivers from the south of Portugal [42,43]. The pH, percentage and ppm of dissolved oxygen and temperature were recorded before and after the experiments to ensure consistency through the two days of experimentation.

For each replicate, the first 10 cm of the track are closed off from the rest with the plastic divider. An individual was then transferred by hand into this enclosed portion for 60 s to acclimatise to the new environment. After 60 s, the divider was removed to allow the individual to roam freely for 120 s, during which the total distance travelled was recorded. If a crayfish reached the opposite end of the track before the 120 ended, we noted the time it took to reach the end and ended the replicate. Crayfish were observed and filmed using a webcam positioned above the steel track. Researchers stood behind a camouflage-patterned cloth to prevent visual contact of crayfish with humans. Motor activity of individuals was assessed by calculating their mean velocity using the total distance travelled. The distances travelled were determined by direct human observation through live camera feed with the aid of measurement markings on the track.

### 2.4. Statistical Analysis

All statistical analyses were done using RStudio (version 4.5.1-2025-06-13 ucrt, Posit Software, PBC, Boston, MA, USA) with the libraries tidyverse, car, lme4, rstatix, pwr and fitdistrplus. We used a repeated measures lm (function lmer() from the lme4 package) to compare the four drug treatments against the control group treatment as well as compare the interactions between treatments with the day, sex and size. We did not test egg carrying as a factor. Velocity values underwent a log(x + 1) transformation to normalise the data and fulfil the homoscedasticity assumption required for parametric statistical analysis (Supplementary Files).

We tested several models, half containing the berried females and the other half without them. The model we chose for this study contains 7 berried females unevenly distributed between the treatments. This model was ultimately chosen because it had a larger sample size and more even distribution of crayfish overall, and although the coefficient of determination was slightly higher in a model without the berried females (Table A2), the model chosen possesses higher size effect values overall (Table A3).

Normal distribution (descdist() from the fitdistrplus library), homoscedasticity (leveneTest() from the car package), statistical power (pwr.f2.test() from the pwr library) and size effect (partial_eta_squared() from the rstatix library) of data were checked for and confirmed. An a priori sample size estimation was conducted using the pwr.f2.test() function from the pwr library to detect an effect size of 0.10, a significant *p*-value of 0.05 and a desired statistical power of 0.80. This estimation recommended a sample size of *n* = 120 per group treatment, considerably bigger than what we were able to use, due to time and budget constraints. With our *n* = 29, statistical power is 0.27 according to the same function.

## 3. Results

Overall, crayfish velocity was not significantly different across the various treatments when compared against the control group, nor between days, sex or sizes (Table 1). The model’s coefficient of determination demonstrates reasonable predictive accuracy for ecological purposes. Adjusted eta-squared analysis also shows strong effect sizes for all variables tested (Table A2).

Regarding remaining models, with or without berried females, none showed significant differences.

## 4. Discussion

The role of fluoxetine, and by extension that of serotonin, on mobility can be of great importance to ecology, especially concerning how exogenous chemical compounds may modulate the locomotion of invasive species. Predicting which species will be the best candidate invaders has been a long-standing dilemma and of increasing interest to ecologists [44,45], and their ability to disperse may be a key factor in determining invasion outcomes as well as the impact and rate of spread [46,47]. Here we exposed *P. clarkii* individuals to 16 days of fluoxetine (2 dosages) as well as para-chlorophenylalanine (pCPA, 2 dosages) and a control to test the effects of changing serotonin levels on their velocity outcome.

Overall, we found that the individual velocity levels did not differ significantly between the treatments and control group, which differs from previous studies on fish that obtained noticeable differences with concentrations of fluoxetine, comparable to those used in this study. For example, Meijide et al. [20] observed a decrease in swimming behaviour of *Gambusia holbrooki* at concentrations of 25 μg/L and 50 μg/L, and Pan et al. [25] reported an increase in distance travelled and faster swimming speeds in *Danio rerio* larvae exposed to 100 μg/L of fluoxetine. However, *P. clarkii* is known to tolerate polluted environments, being capable of accumulating heavy metals and toxins [48] and having higher resistance to drugs such as maduramicin [49]. Moreover, antagonists, such as pCPA, generally need to be administered in larger doses that exceed what is normally required to overcome the agonist effects of the biomolecule they are trying to counteract [50]. The dosages used in our study may have been too low to elicit more conspicuous responses. Furthermore, drug effects can vary due to magnitude and duration of exposure, affecting organisms differently depending on exposure dynamics. For instance, studies have found that either decreases or increases in some behavioural variables such as aggression in crustaceans exposed to serotonin or SSRIs are dependent on the dose and timescale of exposure [51]. This may occur due to stress mediation, which in crayfish happens in connection with the serotonergic system [7,8]. For instance, Fossat et al. [8] showed that stressed *Procambarus clarkii* expressed anxiety-like behaviour that was modulated by 5HT. Serotonin is linked with increased stress levels in crayfish [7], which could have impacted their mobility. If exposed to higher concentrations or longer exposures, we may have seen noticeable alterations to their mobility. Indeed, some studies done on other crustacean species reported fluoxetine influencing locomotion using concentrations higher than those used in this study (i.e., Mesquita et al. [23]; Tierney et al. [1]). Alternatively, in a greater dosage of fluoxetine and pCPA, there could have been an eliciting a shutting down response (aka negative feedback), which may have measurable physiological consequences, but that was not distinguishable in their mobility.

Another explanation as to why there was no measured response could be low uptake of the two substances into the crayfish tissues. Gills were probably the primary site for pharmaceutical uptake given the emersion method we used. Other studies demonstrate that gill absorption of SSRI can produce noticeable effects in crustaceans [52] even at lower concentrations than those we used. However, we did not take tissue or haemolymph samples for our study. Because of this, we could not determine the concentrations of fluoxetine and pCPA in crayfish tissues, nor measure serotonin levels, its metabolites and the expression of the corresponding receptors. The lack of differences in the treatments could be a result of the two pharmaceuticals not being absorbed or due to these being broken down faster than can be taken up.

Also, of importance is that we only used adults that originated from a creek adjacent to rice fields subjected to potential fertiliser and pesticide runoffs, which may even be more resistant to serotonergic drugs. In the wild, organisms would be constantly exposed to pharmaceuticals throughout their lives. Not only could chronic exposure alter the behaviour of crayfish differently than acute exposure would, but different life stages could also be affected differently. Tierney et al. [1] observed that adult *Orconectes rusticus* walking behaviour did not differ significantly from controls when these were exposed to fluoxetine, which led the author to suggest that adult crayfish are more resistant to the behavioural effects of fluoxetine than juveniles are. This too could be the case for *P. clarkii*. Finally, other factors such as temperature variations, the absence or presence of food, change of light intensity and the presence of conspecifics are presently known to be relevant to *P. clarkii* mobility [53]; thus, we assume that these could putatively change the effect of SSRI drugs, as it seemed to affect the spiny-cheek crayfish (*Faxonius limosus*) when exposed to citalopram (see Reisinger et al. [51] or in other experimental contexts (static vs. dynamic systems when exposed to fluoxetine, as seen by Hossain et al. [54].

Fluoxetine mobility effects seem to depend on the species tested. Most of the studies found reductions in distances travelled and the number of times they started moving, as well as delays in starting to move [19,20,55], while a few report an increase in locomotor activity [24,25,26]. Though the exposure period is somewhat comparable to previous studies [1,23], and considering the potential resilience of *P. clarkii*, this exposure period may not have been long enough for fluoxetine to elicit a more significant response. The single behavioural measure considered (velocity) in our study (120 s of reaction to maze, measured on two occasions) may not provide a full range of putative exposure effects. Moreover, we solely conducted measurements on the 8th and 16th day, but there could have been observable changes in the intermediary days that were not measured. Beyond this, a longer exposure period, possibly measured in months rather than weeks, could also elicit noticeable effects.

Regarding limitations, though the experimental design we used has been affected by inaccurate movement observation and measurement in the past by Marques et al. [56], the procedure used has limitations that could have skewed the results. First of all, the sample size used for this study was low, at 28 to 29 crayfish depending on the treatment. Power analysis recommended a sample size of approximately 120 individuals per treatment to achieve a statistical power of 0.8, meaning this study had less than a quarter of the necessary individuals for a strong analysis, potentially skewing the study. Crayfish were manually picked up and moved to the starting end of the track, an action which would have stressed the individuals and affected their locomotion. The 60 s of acclimation and subsequent 120 s of free roaming could have been too short of a time period to allow the crayfish to recover from a potentially stressful situation and to observe a meaningful reaction during free roam. Though total distance travelled was recorded, the number of times they turned, started to move or paused, among other metrics, was not, and as mentioned above, we did not take tissue or haemolymph samples to measure relevant neurochemicals and metabolites. Moreover, we only tested adults from one location, limiting our observations to only one population. On top of this, crayfish were captured in two different seasons and housed for different periods, one group for almost 4 months and the other for only about 2 weeks. These differences could have influenced their physiological state and behaviour. Only two doses of each substance were tested, between which there is a gap of two orders of magnitude. Not only are these concentrations considerably above ecologically relevant levels, but intermediate concentrations are also missing. As previously mentioned, these two concentrations were chosen with the expectation that they would produce a noticeable change for us to measure the putative effects of fluoxetine.

Though observing a wide range of behaviour metrics, dosages and sample sizes were beyond the scope of this study due to time and financial constraints, future research should use a revised set-up with an extended replicate duration, allowing sufficient time for crayfish to adjust and move in the track environment. Subsequent studies should test a wider range of concentrations that match the reality of environmental variations, on a larger sample size that contains crayfish from multiple populations and include significantly more complex measurements of behavioural and motor activity, including sociality, boldness and risk. Additionally, tissue and haemolymph samples should be collected and analysed. This would allow us to better extrapolate the effects that the serotonergic system has on crayfish locomotion while keeping a solid experimental design.

## 5. Conclusions

Our findings show that, overall, fluoxetine and pCPA did not alter *Procambarus clarkii* locomotion in the tested exposure periods and dosages. It is possible that this might be due to crayfishs’ high tolerance to these drugs, although, due to the limited scope of our study, our conclusions remain speculative. Though we believe these findings can be useful in understanding how exposure to ambient fluoxetine affects *P. clarkii* locomotor activity, further testing with an improved experimental design would give us an even better outlook. Faced with current global changes and habitat alterations, it is important to continuously expand our knowledge and understanding of how the presence of pharmaceuticals affects the biology of aquatic organisms. Doing so can prove instrumental in formulating new plans and strategies to mitigate the impacts of invasive species such as *P. clarkii*.

## Figures and Tables

**Table 1:** Repeated Measures LM results table comparing treatments, day, sex, size (cephalothorax length in mm) and the interactions between these. Results obtained from comparing the four drug treatments against the control group using log(x + 1) transformed velocity values; significant *p*-values are displayed in bold (significant at 0.05).

Predictors	Estimates	Std. Error	t Value	*p*	Predictors	Estimates	Std. Error	t Value	*p*
(Intercept)	0.62	0.42	1.49	0.137	pCPA 100 µg × Size	−0.00	0.01	−0.09	0.927
Fluoxetine 1 µg	0.08	0.57	0.14	0.885	Day × Size	0.01	0.01	1.54	0.124
Fluoxetine 100 µg	−0.42	0.60	−0.71	0.481	Sex × Size	0.01	0.01	1.47	0.143
pCPA 1 µg	−0.40	0.54	−0.75	0.451	Fluoxetine 1 µg × Day × Sex	0.19	0.32	0.59	0.558
pCPA 100 µg	0.12	0.61	0.20	0.842	Fluoxetine 100 µg × Day × Sex	0.09	0.34	0.26	0.798
Day	−0.38	0.24	−1.61	0.108	pCPA 1 µg × Day × Sex	−0.33	0.30	−1.09	0.279
Sex	−0.62	0.42	−1.48	0.140	pCPA 100 µg × Day × Sex	−0.03	0.35	−0.08	0.933
Size	−0.01	0.01	−0.81	0.419	Fluoxetine 1 µg × Day × Size	−0.01	0.01	−0.95	0.343
Fluoxetine 1 µg × Day	0.35	0.32	1.11	0.270	Fluoxetine 100 µg × Day × Size	0.00	0.01	0.02	0.983
Fluoxetine 100 µg × Day	−0.01	0.34	−0.03	0.975	pCPA 1 µg × Day × Size	−0.01	0.01	−1.60	0.111
pCPA 1 µg × Day	0.50	0.30	1.64	0.103	pCPA 100 µg × Day × Size	−0.01	0.01	−1.67	0.096
pCPA 100 µg × Day	0.58	0.35	1.68	0.095	Fluoxetine 1 µg × Sex × Size	0.00	0.01	0.05	0.962
Fluoxetine 1 µg × Sex	0.02	0.57	0.04	0.972	Fluoxetine 100 µg × Sex × Size	−0.01	0.01	−0.77	0.442
Fluoxetine 100 µg × Sex	0.47	0.60	0.78	0.435	pCPA 1 µg × Sex × Size	−0.00	0.01	−0.24	0.809
pCPA 1 µg × Sex	0.20	0.54	0.36	0.716	pCPA 100 µg × Sex × Size	−0.01	0.01	−0.97	0.331
pCPA 100 µg × Sex	0.57	0.61	0.94	0.348	Day × Sex × Size	−0.00	0.01	−0.34	0.735
Day × Sex	0.09	0.24	0.39	0.694	Fluoxetine 1 µg × Day × Sex × Size	−0.00	0.01	−0.64	0.524
Fluoxetine 1 µg × Size	−0.00	0.01	−0.13	0.897	Fluoxetine 100 µg × Day × Sex × Size	−0.00	0.01	−0.23	0.821
Fluoxetine 100 µg × Size	0.01	0.01	0.68	0.498	pCPA 1 µg × Day × Sex × Size	0.01	0.01	1.16	0.247
pCPA 1 µg × Size	0.01	0.01	0.86	0.392	pCPA 100 µg × Day × Sex × Size	0.00	0.01	0.08	0.939
Observations	288								
Marginal R^2^/Conditional R^2^	0.152/0.589								

**Figure A1:**
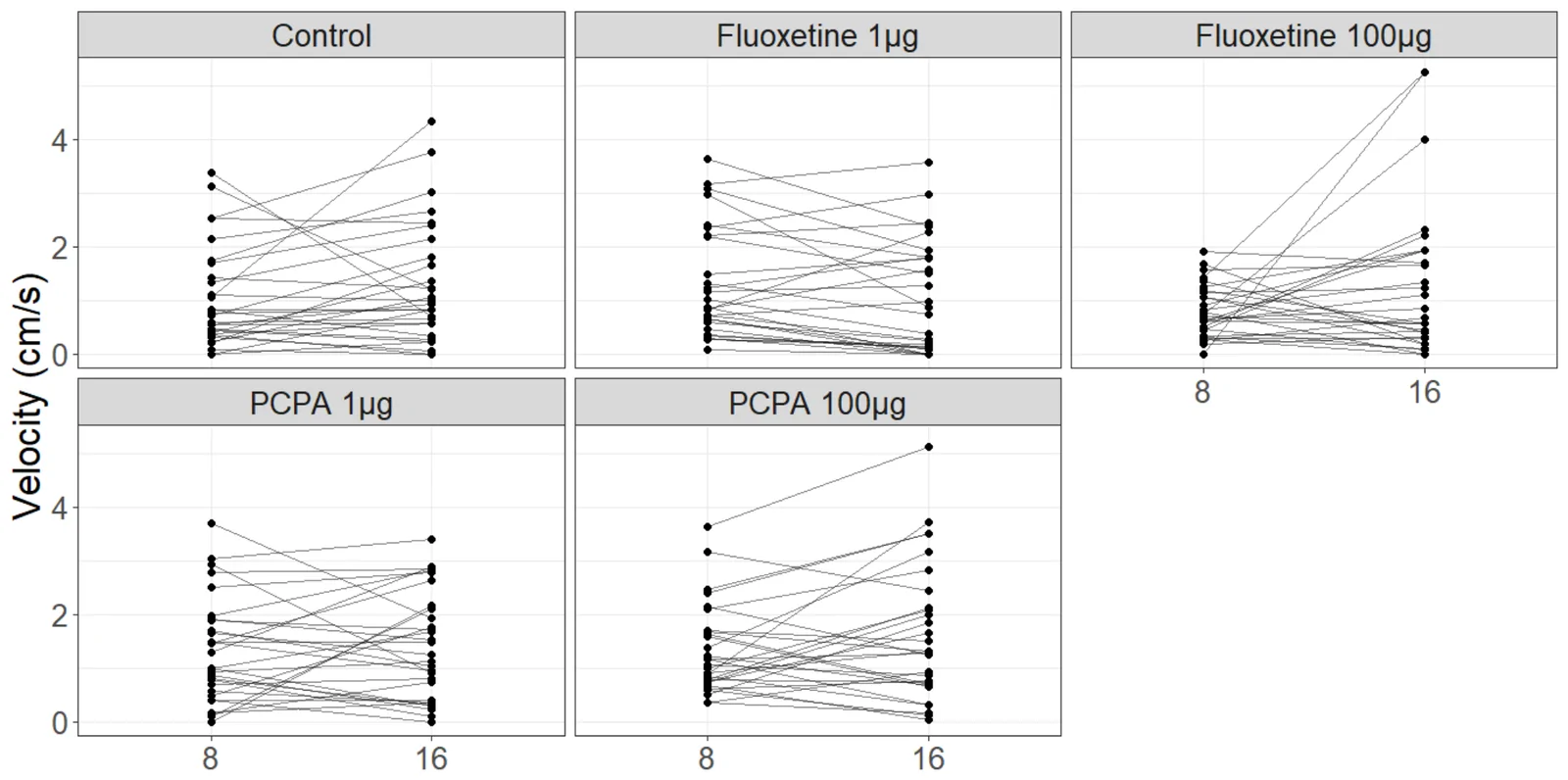
Individual line trajectories graph displaying the velocity variation of crayfish between day 8 and 16.

**Figure A2:**
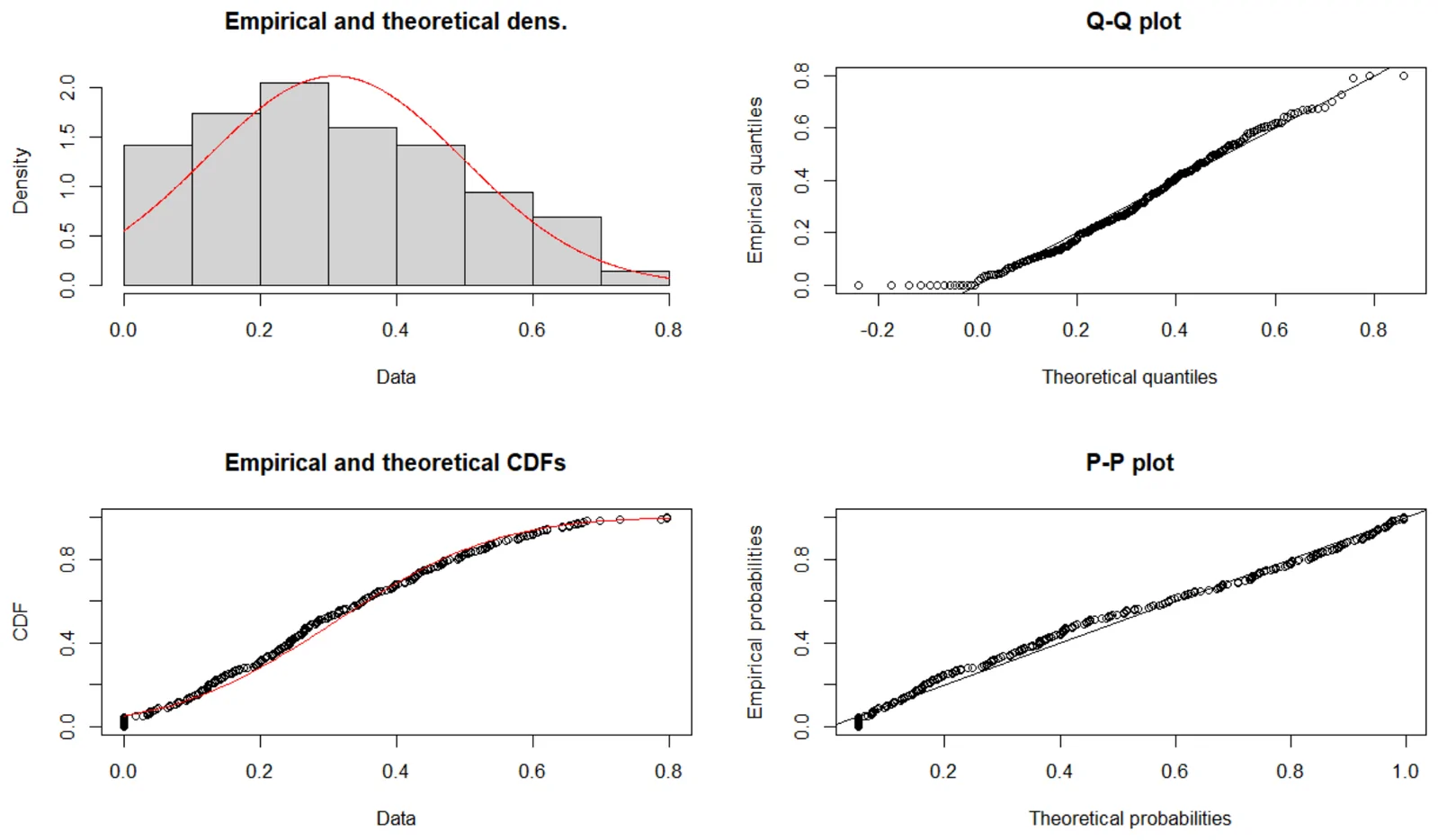
Empirical and theoretical density plot, density Q-Q plot, cdf plot and P-P plot demonstrating goodness-of-fit estimation of the log transformed velocity values for normal distribution.

## Data Availability

The raw data supporting the conclusions of this article will be made available by the authors on request.

## Supplementary Materials

The following supporting information can be downloaded at https://www.mdpi.com/article/10.3390/biology15181670/s1, File ‘Data Chem_Revised.csv’: Raw data file; File ‘DC_revised_V2’: R file containig the scripts for the statistical analysis and tables/graphs.

## Abbreviations

The following abbreviations are used in this manuscript:

SSRI	Selective serotonin reuptake inhibitor
5HT	5-hydroxytryptamine
pCPA	Para-chlorophenylalanine

## Appendix A

**Table A1:** Table of water parameters recorded at the start and end of the four experiment days.

1º Set of Crayfish				2º Set of Crayfish			
Date	21 July 2023			Date	11 August 2023		
Parameter	Start	End	Average	Parameter	Start	End	Average
mV pH	−19.2	−60.8	−40	mV pH	−34.9	−39.4	−37.15
pH	7.42	8.17	7.795	pH	7.7	7.78	7.74
mV ORP	41.7	14.1	27.9	mV ORP	36.3	9.3	22.8
%DO	56.9	87.8	72.35	%DO	46.3	32.4	39.35
ppmDO	4.84	7.4	6.12	ppmDO	4	2.74	3.37
µS/cm	654	831	742.5	µS/cm	784	378	581
µS/cm^A	618	789	703.5	µS/cm^A	738	363	550.5
MΩ × cm	0.0015	0.0012	0.00135	MΩ × cm	0.0013	0.0026	0.00195
ppm Tds	327	415	371	ppm Tds	394	190	292
PSU	0.32	0.41	0.365	PSU	0.39	0.18	0.285
σt	0	0	0	σt	0	0	0
°C	22.13	22.39	22.26	°C	21.89	22.85	22.37
psi	14.36	14.356	14.358	psi	14.437	14.455	14.446
Date	29 July 2023			Date	19 August 2023		
Parameter	Start	End	Average	Parameter	Start	End	Average
mV pH	−28.4	−31.4	−29.9	mV pH	−62.6	−58.2	−60.4
pH	7.58	7.64	7.61	pH	8.2	8.12	8.16
mV ORP	35.6	14.7	25.15	mV ORP	10.2	14.2	12.2
%DO	75.2	85	80.1	%DO	32	34.6	33.3
ppmDO	6.42	7.1	6.76	ppmDO	2.7	2.89	2.795
µS/cm	688	923	805.5	µS/cm	707	662	684.5
µS/cm^A	656	889	772.5	µS/cm^A	678	639	658.5
MΩ × cm	0.0015	0.0011	0.0013	MΩ × cm	0.0014	0.0015	0.00145
ppm Tds	344	461	402.5	ppm Tds	354	328	341
PSU	0.34	0.45	0.395	PSU	0.34	0.32	0.33
σt	0	0	0	σt	0	0	0
°C	22.5	23.03	22,765	°C	22.87	23.23	23.05
psi	14.352	14.37	14.361	psi	14.421	14.422	14.4215

**Table A2:** Coefficient of Determination values of the models tested, with and without berriedfemales included in the dataset. In bold: formula/model chosen for this study.

Formula (With berried females)	n/observations	Marginal R2	Conditional R2
**V1 ~ Treatment * Day * Size * Sex + (1|Individual)**	144/288	0.152	0.589
V1 ~ Treatment * Day * Size + (1|Individual)		0.081	0.559
V1 ~ Treatment * Day * Sex + (1|Individual)		0.084	0.553
V1 ~ Treatment * Size * Sex + (1|Individual)		0.116	0.554
V1 ~ Treatment * Day + (1|Individual)		0.043	0.531
V1 ~ Treatment * Sex + (1|Individual)		0.060	0.527
V1 ~ Treatment * Size + (1|Individual)		0.056	0.530
V1 ~ Treatment + (1|Individual)		0.029	0.515
Formula (Without berried females)	n/observations	Marginal R2	Conditional R2
V1 ~ Treatment * Day * Size * Sex + (1|Individual)	137/274	0.168	0.590
V1 ~ Treatment * Day * Size + (1|Individual)		0.090	0.566
V1 ~ Treatment * Day * Sex + (1|Individual)		0.098	0.555
V1 ~ Treatment * Size * Sex + (1|Individual)		0.133	0.557
V1 ~ Treatment * Day + (1|Individual)		0.040	0.537
V1 ~ Treatment * Sex + (1|Individual)		0.074	0.529
V1 ~ Treatment * Size + (1|Individual)		0.062	0.533
V1 ~ Treatment + (1|Individual)		0.024	0.517

**Table A3:** Partial Eta Squared values for the chosen repeated measures linear model. Left side: model with berried females included in the dataset; Right side: model without berried females in the dataset.

Predictors (With berried females)	η^2^p	Interpretation
Treatment	0.302	Strong Correlation
Day	0.179	Strong Correlation
Sex	0.492	Strong Correlation
Size	0.209	Strong Correlation
Treatment × Day	0.569	Strong Correlation
Treatment × Sex	0.263	Strong Correlation
Day × Sex	0.116	Moderate Correlation
Treatment × Size	0.306	Strong Correlation
Day × Size	0.167	Strong Correlation
Sex × Size	0.515	Strong Correlation
Treatment × Day × Sex	0.468	Strong Correlation
Treatment × Day × Size	0.559	Strong Correlation
Treatment × Sex × Size	0.295	Strong Correlation
Day × Sex × Size	0.077	Moderate Correlation
Predictors (Without berried females)	η^2^p	Interpretation
Treatment	0.314	Strong Correlation
Day	0.100	Moderate Correlation
Sex	0.518	Strong Correlation
Size	0.107	Moderate Correlation
Treatment × Day	0.590	Strong Correlatio
Treatment × Sex	0.285	Strong Correlation
Day × Sex	0.162	Strong Correlation
Treatment × Size	0.307	Strong Correlation
Day × Size	0.090	Moderate Correlation
Sex × Size	0.548	Strong Correlation
Treatment × Day × Sex	0.482	Strong Correlation
Treatment × Day × Size	0.582	Strong Correlation
Treatment × Sex × Size	0.324	Strong Correlation
Day × Sex × Size	0.119	Moderate Correlation
